# The COVID-19 Data Portal: accelerating SARS-CoV-2 and COVID-19 research through rapid open access data sharing

**DOI:** 10.1093/nar/gkab417

**Published:** 2021-05-28

**Authors:** Peter W Harrison, Rodrigo Lopez, Nadim Rahman, Stefan Gutnick Allen, Raheela Aslam, Nicola Buso, Carla Cummins, Yasmin Fathy, Eloy Felix, Mihai Glont, Suran Jayathilaka, Sandeep Kadam, Manish Kumar, Katharina B Lauer, Geetika Malhotra, Abayomi Mosaku, Ossama Edbali, Young Mi Park, Andrew Parton, Matt Pearce, Jose Francisco Estrada Pena, Joseph Rossetto, Craig Russell, Sandeep Selvakumar, Xènia Pérez Sitjà, Alexey Sokolov, Ross Thorne, Marianna Ventouratou, Peter Walter, Galabina Yordanova, Amonida Zadissa, Guy Cochrane, Niklas Blomberg, Rolf Apweiler

**Affiliations:** European Molecular Biology Laboratory, European Bioinformatics Institute, Wellcome Genome Campus, Hinxton, Cambridge CB10 1SD, UK; European Molecular Biology Laboratory, European Bioinformatics Institute, Wellcome Genome Campus, Hinxton, Cambridge CB10 1SD, UK; European Molecular Biology Laboratory, European Bioinformatics Institute, Wellcome Genome Campus, Hinxton, Cambridge CB10 1SD, UK; European Molecular Biology Laboratory, European Bioinformatics Institute, Wellcome Genome Campus, Hinxton, Cambridge CB10 1SD, UK; European Molecular Biology Laboratory, European Bioinformatics Institute, Wellcome Genome Campus, Hinxton, Cambridge CB10 1SD, UK; European Molecular Biology Laboratory, European Bioinformatics Institute, Wellcome Genome Campus, Hinxton, Cambridge CB10 1SD, UK; European Molecular Biology Laboratory, European Bioinformatics Institute, Wellcome Genome Campus, Hinxton, Cambridge CB10 1SD, UK; European Molecular Biology Laboratory, European Bioinformatics Institute, Wellcome Genome Campus, Hinxton, Cambridge CB10 1SD, UK; European Molecular Biology Laboratory, European Bioinformatics Institute, Wellcome Genome Campus, Hinxton, Cambridge CB10 1SD, UK; European Molecular Biology Laboratory, European Bioinformatics Institute, Wellcome Genome Campus, Hinxton, Cambridge CB10 1SD, UK; European Molecular Biology Laboratory, European Bioinformatics Institute, Wellcome Genome Campus, Hinxton, Cambridge CB10 1SD, UK; European Molecular Biology Laboratory, European Bioinformatics Institute, Wellcome Genome Campus, Hinxton, Cambridge CB10 1SD, UK; European Molecular Biology Laboratory, European Bioinformatics Institute, Wellcome Genome Campus, Hinxton, Cambridge CB10 1SD, UK; ELIXIR, Wellcome Genome Campus, Hinxton, Cambridge CB10 1SD, UK; European Molecular Biology Laboratory, European Bioinformatics Institute, Wellcome Genome Campus, Hinxton, Cambridge CB10 1SD, UK; European Molecular Biology Laboratory, European Bioinformatics Institute, Wellcome Genome Campus, Hinxton, Cambridge CB10 1SD, UK; European Molecular Biology Laboratory, European Bioinformatics Institute, Wellcome Genome Campus, Hinxton, Cambridge CB10 1SD, UK; European Molecular Biology Laboratory, European Bioinformatics Institute, Wellcome Genome Campus, Hinxton, Cambridge CB10 1SD, UK; European Molecular Biology Laboratory, European Bioinformatics Institute, Wellcome Genome Campus, Hinxton, Cambridge CB10 1SD, UK; European Molecular Biology Laboratory, European Bioinformatics Institute, Wellcome Genome Campus, Hinxton, Cambridge CB10 1SD, UK; European Molecular Biology Laboratory, European Bioinformatics Institute, Wellcome Genome Campus, Hinxton, Cambridge CB10 1SD, UK; European Molecular Biology Laboratory, European Bioinformatics Institute, Wellcome Genome Campus, Hinxton, Cambridge CB10 1SD, UK; European Molecular Biology Laboratory, European Bioinformatics Institute, Wellcome Genome Campus, Hinxton, Cambridge CB10 1SD, UK; European Molecular Biology Laboratory, European Bioinformatics Institute, Wellcome Genome Campus, Hinxton, Cambridge CB10 1SD, UK; ELIXIR, Wellcome Genome Campus, Hinxton, Cambridge CB10 1SD, UK; European Molecular Biology Laboratory, European Bioinformatics Institute, Wellcome Genome Campus, Hinxton, Cambridge CB10 1SD, UK; European Molecular Biology Laboratory, European Bioinformatics Institute, Wellcome Genome Campus, Hinxton, Cambridge CB10 1SD, UK; European Molecular Biology Laboratory, European Bioinformatics Institute, Wellcome Genome Campus, Hinxton, Cambridge CB10 1SD, UK; European Molecular Biology Laboratory, European Bioinformatics Institute, Wellcome Genome Campus, Hinxton, Cambridge CB10 1SD, UK; European Molecular Biology Laboratory, European Bioinformatics Institute, Wellcome Genome Campus, Hinxton, Cambridge CB10 1SD, UK; European Molecular Biology Laboratory, European Bioinformatics Institute, Wellcome Genome Campus, Hinxton, Cambridge CB10 1SD, UK; European Molecular Biology Laboratory, European Bioinformatics Institute, Wellcome Genome Campus, Hinxton, Cambridge CB10 1SD, UK; ELIXIR, Wellcome Genome Campus, Hinxton, Cambridge CB10 1SD, UK; European Molecular Biology Laboratory, European Bioinformatics Institute, Wellcome Genome Campus, Hinxton, Cambridge CB10 1SD, UK

## Abstract

The severe acute respiratory syndrome coronavirus 2 (SARS-CoV-2) pandemic will be remembered as one of the defining events of the 21st century. The rapid global outbreak has had significant impacts on human society and is already responsible for millions of deaths. Understanding and tackling the impact of the virus has required a worldwide mobilisation and coordination of scientific research. The COVID-19 Data Portal (https://www.covid19dataportal.org/) was first released as part of the European COVID-19 Data Platform, on April 20th 2020 to facilitate rapid and open data sharing and analysis, to accelerate global SARS-CoV-2 and COVID-19 research. The COVID-19 Data Portal has fortnightly feature releases to continue to add new data types, search options, visualisations and improvements based on user feedback and research. The open datasets and intuitive suite of search, identification and download services, represent a truly FAIR (Findable, Accessible, Interoperable and Reusable) resource that enables researchers to easily identify and quickly obtain the key datasets needed for their COVID-19 research.

## Graphical Abstract

**Graphical Abstract:**
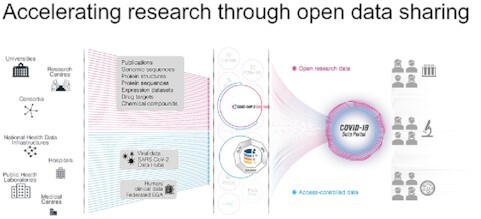
The Graphical Abstract depicts the flow of COVID-19 related information from independent data resources and their consolidation into a single integrated system.

## INTRODUCTION

**Figure 1.**
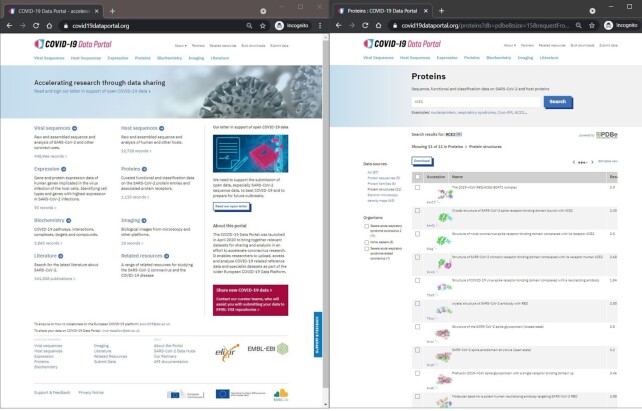
shows the home page of the https://www.covid19dataportal.org portal and results from a search for ACE2 on the Protein structures category on the Proteins section.

The COVID-19 Data Portal (CDP; https://www.covid19dataportal.org/) aims to accelerate global SARS-CoV-2 research through rapid open access data sharing (see Figure 1). The CDP forms part of the larger European COVID-19 Data Platform (1) that also comprises the SARS-CoV-2 Data Hubs and the Federated European Genome-Phenome Archive (EGA) (2). The CDP is developed by a task force from the European Molecular Biology Laboratory's European Bioinformatics Institute (EMBL-EBI; https://www.ebi.ac.uk/).

The CDP provides coverage of both SARS-CoV-2 virus – in particular its ∼30 kb RNA genome and 29 genes (https://cen.acs.org/biological-chemistry/infectious-disease/know-novel-coronaviruss-29-proteins/98/web/2020/04)—and its human host. In addition, data are available for non-SARS-CoV-2 coronaviruses and non-human hosts. The resource spans biomolecular data, comprising experimental and computational data types related to the genome, genes, proteins of SARS-CoV-2, structural and biochemical interactions, and literature available from EMBL-EBI and ELIXIR (https://elixir-europe.org/) data resources.

## DATASETS AND AVAILABILITY

The CDP consists of seven main data categories: viral sequences, host sequences, expression, proteins, biochemistry, imaging and literature. Here, we describe the data types available in each of these sections.

### Viral sequences

From the early days of the COVID-19 pandemic, nucleotide sequencing data has been deposited in the European Nucleotide Archive (ENA; https://www.ebi.ac.uk/ena/browser/home) (3). This has led to a surge in raw and assembled nucleotide sequences (as depicted in graphs on the statistics page of the CDP https://www.covid19dataportal.org/statistics), COVID-19 Data Portal: https://www.covid19dataportal.org/statistics), owing in part to dedicated viral data mobilisation efforts. The Viral sequences data category includes publicly shared SARS-CoV-2 whole genomes (sequences), presenting metadata such as date of sample collection and geographic location, which allows for the characterisation of these data at the national and institutional levels. Alongside SARS-CoV-2 genomes, raw sequencing reads have also been submitted, which correlate to sequenced SARS-CoV-2 biological samples from human hosts from 41 countries across the world. With early sharing of the SARS-CoV-2 reference genome, genes comprising the virus have been annotated through Ensembl (4) and are available from the Gene category. These are also available through the Ensembl COVID-19 genome browser (https://covid-19.ensembl.org/SARS_cov_2/Info/Index). Variation within the SARS-CoV-2 genome has been captured through variant calling of Nextstrain and ENA data, processed with Ensembl and available in the Variants category. Refinements on variation calling and presentation are underway.

### Host sequences

Access to sensitive, human raw sequencing and biomolecular data, including data from COVID-19 patient and research subject studies, shared with authorised access through the EGA, are available under the Host sequences section in the CDP. Authorised access to these datasets is provided through Data Access Committees according to established EGA processes. SARS-CoV-2 related genome-wide association studies from the GWAS Catalogue (5) reside in this category and provide access to datasets from studies looking into genetic variations in contexts such as susceptibility and severity of COVID-19 and its spread. Non-sensitive human raw sequencing data from blood and tissue cell studies containing SARS-CoV-2 are archived in the ENA and available for download directly from the CDP. In addition, raw sequencing data of other non-human host species, for example from mouse studies, are also available under the Host sequences section.

### Expression

Under the Expression data category, expression datasets and experiments for host genes and proteins involved in human SARS-CoV-2 transmission and infection are made available. These datasets are provided through the Expression Atlas (6) and the Proteomics Identifications Database (PRIDE) (7). The Single Cell Expression Atlas provides cell and cell type datasets with elevated expression. These include experiments in human and mouse tissues that include time track and infection profiles.

### Proteins

Protein sequences from UniProtKB (8) that represent human gene products with experimental evidence of interaction with SARS-CoV-2 surface proteins, as well as with potential compound targets and models, are accessible in the Protein section of the Data Portal. Also found in this section are protein structures from the PDBe-KB data resource (9) and PDBe (10) that highlight important structural features, including ligand binding sites and protein-protein interaction residues important in the research and development of vaccines and therapeutics against SARS-CoV-2. Protein families from InterPro (11) include entries that match any of the virus proteins and that help with further identification and characterisation of protein variants. The Electron microscopy maps and images category contains entries from The Electron Microscopy Databank (12) and The Electron Microscopy Public Imaging Archive. This includes data from EMPIAR (13) that provides access to 3-D structural models of the S-protein trimer, which is critical in the design of vaccines, and other virus proteins that may have potential to serve as antigens or compound targets.

### Biochemistry

Biomolecular interactions, targets, compounds, complexes and pathways implicated in COVID-19 comprise the Biochemistry section of the portal. Biological pathways identified in SARS-CoV-2 host cell infection and replication from Reactome (14) are available here, as well as molecular interaction data from user submissions and literature curation from the IntAct Molecular Interaction Database (15). It also provides access to macromolecular complexes from the Complex Portal (16), the majority of which are protein complexes involved in processes facilitating virus entry, replication and spread.

Furthermore, studies investigating compounds and their effects on SARS-CoV-2 activity (for example studies researching potential therapeutic targets), are available from the Compound documents category, which presents data from ChEMBL (17). Open Targets (18) provides evidence of drug targets activity against COVID-19 on the Drug targets category. MetaboLights (19) entries relating to SARS-CoV-2 infection and metabolic effects are available through the Metabolomics experiments category.

### Imaging

This section contains data from BioStudies (20) providing data on compound screening and assays, as well as archived microscopic images for SARS-CoV-2 studies. Electron microscopy imaging details data is also available through EMPIAR, characterising the virus structures and its infection in various human tissues. These datasets are also accessible through the Biochemistry section.

### Literature

The CDP includes extensive automated literature detailing SARS-CoV-2, COVID-19 and its effects, and related viruses and diseases present through Europe PMC (21). The utilisation of prepublication resources, such as bioRxiv, have become key for a rapid dissemination route for SARS-CoV-2 research. Europe PMC has indexed these key prepublication COVID-19 manuscripts to ensure visibility in this rapidly evolving field. The Literature section is solely dedicated to making these publications available to the user community.

## RELATED RESOURCES

Beyond the fine-grained biomolecular and literature access available from the services described above, a number of further biomolecular data and other resources are provided under the Related resources section of the CDP. Here, top-level information and links to relevant entry points are provided to databases, tools and catalogues of relevance to COVID-19. At the time of writing, 70 resources are linked.

## DOCUMENTATION AND GUIDELINES

The CDP includes a number of pages providing information on the European COVID-19 Data Platform as a whole, data submission tools, data standards of relevance and partners and funded projects involved in the Platform's operations.

## IMPORTANCE OF OPEN ACCESS DATA SHARING

Open science and open data are principles that are shared by all EMBL-EBI and ELIXIR data resources and, from its inception, the CDP has had the facilitation of open data sharing as a core concept. It was recognised that unrestricted access to data plays a critical role in the rapid coronavirus research necessary to respond to this global health crisis. This is crucial for the identification of drug targets, developing vaccines, understanding infection and symptoms, tracking the effects of new variants, and for policy makers designing public health responses. Ensuring open science and unrestricted international collaborations is of key importance, and it is recognised that these datasets must be shared openly and meet FAIR standards (Findable, Accessible, Interoperable and Reusable).

To this end, the CDP recently hosted an open letter (https://www.covid19dataportal.org/support-data-sharing-covid19) jointly calling upon the data submitters, data users, policy makers and the wider research community, to support truly open and unrestricted data sharing. At the time of writing this letter has attracted 764 signatures. The letter calls for submission of raw SARS-CoV-2 data to the databases of the International Nucleotide Sequence Database Collaboration (INSDC) (22). Authors ask submitters to provide a key minimum set of information relating to the sequenced isolate or sample as part of the sequence submission, to maximise the value of the sequences. Promoting open data sharing will also be key for future pandemics, and to unleash the fast flow of research advances into clinical use of sequencing for the benefit of society.

## COMPONENTS AND APIs

The CDP provides a categorised collection of related resources including complementary databases, analysis tools, data standards, related European projects and connections to ELIXIR resources.

Searching and identifying relevant datasets for COVID-19 research is one of the challenges the CDP meets head on. EMBL-EBI has invested in the development and maintenance of a scalable text search engine, EBI Search (23), that is currently indexing >3.7 billion records, comprising the majority of data resources available from the institute. The data in the CDP is obtained using the EBI Search API, making use of its advanced search functionality. For virus sequences, the EBI Search API and thus the CDP is capable of querying genes, targets, sequencing technology, isolate or strain names, submission centre names, country and protein products. The data retrieved can then be easily sorted by submission or collection date.

Data obtained from the ENA is divided into data type sections, denoting sequences, reference sequences, raw reads, sample, studies, genes, browser assemblies and variants. These data can be searched, for example, by accession number, date and date range, country, strains, isolates, hosts and sequencing centre name. To help the user navigate these data there are multi-selectable facets, for example, organism, first public date, collection date and sequencing centre name.

Controlled-access human data come from serology antibody studies, immunoprofiling, paired-end sequencing and genome wide genotyping. It is important to stress that these data are in the public domain and have been anonymised by the submitters. These data can be searched by study identifier (for example, EGAS00001004412) or by using free text descriptions.

Gene and protein expression experiments can be queried by the gene name, organ or tissue and disease association. Methods used to collect samples, such as nasal swabs, can also be used to query, although they often appear as part of free text fields in the data. Queries in these resources include expression accession numbers, gene names, Ensembl identifiers and PRIDE experiment identifiers.

Protein data covers a broad set of queryable fields, including biomolecular structure terms, protein family categories, related organisms, authors, literature titles, dates and data types. For example, the key COVID-19 protein sequences for ACE2 Q9BYF1 or ACE2_HUMAN from UniProtKB; the coronavirus S-protein IPR002551 from InterPro; the spike receptor binding domain complex 6lzj from PDBe; and the SARS-CoV-2 S trimer EMD-30701.

The key user queries that are possible in the Biochemistry section include the names of metabolic pathways related to the innate immune response of the host, maturation of various proteins, translation and replication. The associated compounds literature shows anti-coronavirus activity and drug targets with disease association scores, such as cathepsin and metabolomic experiments showing response to both drugs and infection. For example, MTBLS1987 describes the kynurenic acid sex-specific immune response in COVID-19.

The CDP has a section dedicated to imaging data. At present this comprises studies, such as high content screens of cells treated with compound libraries. It also contains assays for COVID-19 antibodies based on, for example, high throughput microscopy, high resolution electron microscopy, movies of interaction in complexes and cryo-electron microscopy.

Literature queries are possible that have authors, title and publications divided into categories that distinguish results based on criteria such as coronavirus, related viruses, gene, receptor and antibodies, as well as other supplemental data.

Looking for further ways of improving the search and retrieval capabilities of the CDP is always high on the agenda and further feedback is most welcome. The CDP readily accepts feature requests from the community it supports through its inbuilt feedback pages of the CDP mechanism. The CDP has a basic RESTful API page that shows the relevant calls it makes to the various resources, available here: https://www.covid19dataportal.org/api-documentation. Any user can make use of these APIs in a free and unrestricted manner as long as this is in accordance with the EMBL-EBI General Terms of Use.

The CDP supports the direct download of all the sequences in a section, or up to 1000 selected sequences per page. Due to the size of the sequencing datasets we also provide files containing the complete COVID-19 datasets via FTP. For ease of download (particularly on slower internet connections) the files have been split into chunks that can be easily re-combined to form the complete dataset. These files are available using the Aspera's FASP (https://www.ibm.com/uk-en/products/aspera) file transfer protocol that is particularly effective for large downloads over less stable internet connections. This is essential considering the global importance of these datasets and the fact that many researchers are not currently able to work on site at their institutes, due to lockdown restrictions.

The CDP includes automatically generated statistics (https://www.covid19dataportal.org/statistics) that are updated as new data arrives. The charts track the growth of SARS-CoV-2 viral sequences held in the CDP from different countries, sequencing centres and contributing institutions.

## USAGE

Since its launch in April 2020, the CDP has been accessed over 4 million times from 161 000 unique IP addresses from all over the world. At the time of writing, this translates to approximately 70% usage from various Internet Service Providers and the Data Centre hosting sector, which we believe reflects the various lockdowns and remote working during 2020 and 2021. Eleven percent of all IPs are from the commercial sector, including pharmaceutical companies, national and regional health centres and government organisations, and 5% are from hosts in the education sector.

## FUTURE WORK

The CDP has continued to evolve rapidly to meet the growing needs of the highly active global research community. The CDP Task Force makes feature updates to the CDP approximately every two weeks, announcing the feature changes via the EMBL-EBI twitter account (@emblebi). In addition to more data resources and data types being added as COVID-19 annotations become available, plans to implement a cohort browser, genome assembly visualisation and a host of other analyses and visualisations are in the current roadmap.

## DATA AVAILABILITY

All datasets supporting the CDP are available without restrictions from public data repositories.
